# Correlates of sedentary behaviors among adults from eastern Poland

**DOI:** 10.3389/fpubh.2025.1588908

**Published:** 2025-07-22

**Authors:** Marian Jan Stelmach, Joanna Baj-Korpak, Ewelina Anna Niźnikowska, Barbara Bergier, Michał Bergier, Dorota Tomczyszyn, Paulo Rocha

**Affiliations:** ^1^Department of Tourism and Recreation, Faculty of Health Sciences, John Paul II University in Biała Podlaska, Biała Podlaska, Poland; ^2^Department of Physiotherapy, Faculty of Health Sciences, John Paul II University in Biała Podlaska, Biała Podlaska, Poland; ^3^Department of Sociology, Faculty of Health Sciences, John Paul II University in Biała Podlaska, Biała Podlaska, Poland; ^4^Department of Sport, Instituto Português do Desporto e Juventude, Lisbon, Portugal

**Keywords:** physical inactivity, sociodemographics factors, health, eastern Poland, sedentary behavior, adults (MeSH)

## Abstract

**Background:**

Research on sedentary behaviors in the Polish population using objective methods, such as accelerometry, remains limited. These behaviors, defined as time spent on passive activities or minimal physical effort, require further investigation. This study aimed to identify socio-demographic and health-related correlates of sedentary behaviors in a cohort of adults from eastern Poland.

**Methods:**

A total of 173 adults from eastern Poland participated in the study. Socio-demographic data were collected using the EHIS (wave 3) questionnaire. Movement behaviors were monitored for 7 days using a triaxial accelerometer. Statistical analyses focused on the prevalence of sedentary behaviors (SB) and correlations for qualitative and quantitative variables for two- and multiple-group comparisons. The final stage involved regression models explaining SB and step count per day.

**Results:**

Participants spent an average of 8 h and 34 min per day in sedentary behaviors, with a mean daily step count exceeding 8,000. Self-rated health, gender, employment status, and marital status were the strongest correlates of sedentary behavior. Linear regression analysis showed that in the case of step count per day, employment status is a statistically significant predictor explaining 11.8% of the variance.

**Conclusion:**

The obtained findings underscore the necessity for further research to explore the causal relationships of the prevalence of sedentary behaviors, particularly among socially and professionally excluded individuals.

## Introduction

The modern world is grappling with an epidemic of non-communicable chronic diseases (NCDs), which currently account for the majority of health risks. Among these, the most common are cardiovascular diseases, cancers, type 2 diabetes, respiratory diseases, mental disorders, obesity and its complications, as well as musculoskeletal disorders (1–3). The occurrence of these diseases could largely be reduced by increasing health-enhancing physical activity (HEPA) while significantly decreasing sedentary behaviors (SB) (4). We now have an extensive body of scientific evidence gathered over the past approximately 70 years, indicating that a lifestyle incorporating regular, moderate-to-vigorous physical activity (MVPA) can prevent, and in many cases even treat, NCDs (5–8).

The rapid development of technology after World War II, particularly in the areas of work automation, automobile use, and informatization, has led to the widespread prevalence of sedentary behavior (SB) on a large scale. Assessing the prevalence of this phenomenon became possible thanks to innovative research tools such as accelerometers, which were first applied at the turn of the century to monitor movement behaviors. These tools provided a realistic picture of physical activity within studied communities. Research conducted using accelerometers revealed that SB could be a major risk factor for mortality (9, 10), regardless of the level of physical activity (PA) (11). Some authors even equate its impact to that of smoking (12).

Sedentary behaviors, as opposed to physical inactivity – understood as an insufficient amount of physical effort relative to current guidelines – refer to any movement behaviors (MB) performed during daily activities that are characterized by an energy expenditure of no more than 1.5 metabolic equivalents (METs) while in a sitting, reclining, or lying position. These behaviors are distinct from a complete lack of physical activity and simultaneously represent an independent risk factor for NCDs, even if an individual meets the guidelines for the recommended minimum level of physical activity (13).

The increasing daily duration of SB is becoming a growing problem worldwide, both in developed and developing countries. The civilizational advancements of the past 50 years have led to an extension of leisure time, which most people spend passively, often in seated positions. At the same time, a sedentary lifestyle (SLS) is becoming more widespread. This lifestyle is defined as daily activity not exceeding 60 min, dominated by movement behaviors requiring minimal energy expenditure, with most of the time spent sitting and/or lying down and performing light household tasks (14). An indirect indicator of such a lifestyle, as well as a measure of sedentary behavior preferences, is the number of steps taken per day (15, 16).

In industrialized countries, the average person is inactive and spends more than half of the day in a seated position (17). In the United States, among young adults, this proportion exceeds even 60% of daily activity (18). In Europe, despite a halt in the increasing trend of SB between 2002 and 2017 (19, 20), approximately 40% of the population still preferred passive forms of daily activity, particularly during leisure time (21). However, recent analyses of population data indicate a significant increase in time spent sitting among European adults aged 21–65 (22).

In Poland, as in other European countries, a sedentary lifestyle (SLS) dominates the daily structure of movement behaviors (MB). The 2021 report by the Public Health Committee of the Polish Academy of Sciences (23), based primarily on two large representative studies, NATPOL (24) and WOBASZ (25), shows that as many as 82% of Poles over the age of 15 do not meet WHO recommendations for physical activity. It is worth noting, however, that previous studies on sedentary behaviors in the Polish population are predominantly descriptive, focusing on survey-based studies that highlight insufficient levels of physical activity (inactivity). There is a lack of significant research in which sedentary behaviors (understood as time spent on passive activities or minimal physical effort) and their prevalence in the population are measured using objective methods, such as accelerometry. Equally important is the examination of the determinants and correlates of these behaviors based on the assumptions of the socio-ecological theory of behavior (26). Additionally, Polish literature lacks studies that analyze these issues using objective measurements aligned with the definitions proposed by Bauman and co-authors (27, 28).

This study aims to address this research gap regarding the prevalence and correlates of sedentary behaviors among adult residents of eastern Poland using objective measurement methods based on accelerometry. The geographical region where the study was conducted is quite distinctive – sparsely urbanized, predominantly agricultural, yet characterized by a highly walkable environment but with a high rate of a social environment that promotes sedentary behavior.

We hypothesize that objective measurements will reveal the true extent of sedentary behaviors and the socio-demographic correlates influencing movement behavior patterns. The study contributes new, objective data to the literature on sedentary behaviors in the Polish context, providing insights for designing public health interventions aimed at reducing sedentary lifestyles and promoting health-enhancing physical activity. In our view, the results obtained can support the development of targeted interventions to improve the overall health and well-being of residents in eastern Poland.

## Methods

### Study design

This study utilized data collected in Poland as part of the European project EUPASMOS/EUPASMOS-PLUS (https://erasmus-plus.ec.europa.eu/projects/search/details/603328-EPP-1-2018-1-PT-SPO-SCP as of 22.10.2024), which complements the EUPASMOS project (https://erasmus-plus.ec.europa.eu/projects/search/details/590662-EPP-1-2017-1-PT-SPO-SCP as of 22.10.2024) – an international cohort study involving a total of 18 European Union countries (Bulgaria, Cyprus, Finland, France, Greece, Hungary, Italy, Latvia, Lithuania, North Macedonia, Malta, Netherlands, Poland, Portugal, Romania, Slovenia, Spain, Sweden). Observational data collection was conducted from April to mid-July 2019 and from September 2019 to January 2020.

The study was approved by the Ethics Committee of the Pope John Paul II State School of Higher Education in Biala Podlaska (6/2018), and all the procedures were based on the Declaration of Helsinki. All participants provided written informed consent for participation.

### Participants and study procedure

Adults from the town of Biała Podlaska and the Biała Podlaska County in eastern Poland were invited to participate in the study. The selection of groups was done purposively. The inclusion criteria for the study were: (a) submission of a consent form for participation, (b) age 18 or older, (c) no health contraindications to engaging in health-enhancing physical activity. The exclusion criteria included: (a) refusal to participate in the study, (b) age under 18, (c) health conditions that would exclude participation in any physical activities. A total of 173 adult participants of Caucasian descent, both male and female, took part in the study. The selection of the sample was carried out in such a way as to ensure representativeness in terms of gender and age of the subjects, and to allow validation of the measurement methods used (according to COSMIN criteria - https://www.cosmin.nl/). Participants were divided into four age groups: (1) 18–34 years, (2) 35–49 years, (3) 50–64 years, and (4) over 64 years (Table 1).

**Table 1:** Demographic characteristics of the participants in the EUPASMOS plus Poland cohort study.

Characteristics	Category	*N*	%
Gender	Male	102	59.0
	Female	71	41.0
Age group	<35	53	30.6
	35–49	41	23.7
	50–64	44	25.4
	>64	35	20.2
Health status	Very good	29	16.8
	Good	95	54.9
	Fair	48	27.7
	Bad	1	0.6
	Very bad	0	0.0
Area of residence	Big city	1	0.6
	Small town	135	78.0
	Village	37	21.4
Type of housing	Apartment	73	42.2
	House	100	57.8
Household size	1	25	14.5
	2	63	36.4
	3	36	20.8
	4	28	16.2
	5	14	8.1
	6	6	3.5
	8	1	0.6
Marital status	Single	50	28.9
	Married	91	52.6
	Divorced	18	10.4
	Widowed	12	6.9
	Missing	2	1.3
Current employment status	White-collar worker	87	50.3
	Blue-collar worker	1	0.6
	Unemployed	43	24.9
	Student	39	22.5
	Other	3	1.7
	Total	**173**	**100**

Bold values represent the totals of respondents in each socio-demographic category and the sum of percentages.

On the first day, each participant in the study was introduced to the purpose and procedure of the research. After giving written consent to participate, the respondents were interviewed using a questionnaire to collect basic socio-demographic data. They were also trained in how to wear and use the accelerometer. All participants were required to wear the accelerometer for seven consecutive days (24/7) and keep additional research documentation in the form of: (a) a physical activity diary and (b) a report detailing the periods during which the accelerometer was not worn for various reasons within the monitoring period. Each participant was required to visit the research laboratory on the eighth day to download the recorded data and return the measuring devices.

### Survey and anthropometric data collection

Socio-demographic data were collected based on the European Health Interview Survey (EHIS wave 3) questionnaire (29). The survey provided basic information regarding age, gender, education, marital status, and area of residence. Additionally, respondents subjectively assessed their overall health using a 5-point Likert scale included in the questionnaire.

### Accelerometer measurements

Movement behaviors were monitored continuously for seven consecutive days (24/7) using a triaxial accelerometer, model RM42 (UKK, Tampere, Finland), which measures and records acceleration at a sampling frequency of 100 Hz with 13-bit analog-to-digital conversion within a range of ±16 g. The results were analyzed in non-overlapping 5-s epochs, calculating the Mean Amplitude Deviation (MAD) (30). The accelerometer, secured with an elastic belt, was worn during the day on the right side of the hip at the iliac crest, and during sleep, it was worn on the wrist of the non-dominant hand using a strap. The raw accelerometer data were transmitted to the UKK laboratory, where they were processed into numerical data suitable for statistical analysis. We defined sedentary behavior as any waking behavior with an energy expenditure ≤1.5 METs in a sitting, reclining, or lying position, as proposed by Tremblay et al. (31)

### Statistical analysis

The data collected through surveys, anthropometric measurements, and accelerometer recordings were statistically analyzed using IBM SPSS Statistics 29. The conducted statistical analyses allowed for an assessment of the prevalence of sedentary behaviors and an investigation of which socio-demographic and health-related factors, and to what extent, influence sedentary behaviors within the studied cohort. Initially, basic descriptive statistics were computed, along with the Shapiro–Wilk test to check for normality assumptions for the primary variables related to a sedentary lifestyle, such as time spent sitting/lying down (SB) and the number of steps taken during the entire monitoring period. Subsequently, the correlations between ordinal and quantitative variables were assessed using Spearman’s rho correlation analysis. The Mann–Whitney U test was also conducted to compare two unequal groups, and the Kruskal-Wallis test was applied for comparing more than two unequal groups. The choice of non-parametric tests was based on the properties of the data that were analyzed. The normality tests indicated that the data were not normally distributed, and that certain variables, such as socio-economic status, were measured on an ordinal scale. This meant that it was not possible to use parametric tests. For all comparative analyses, a significance level of *α* = 0.05 was adopted.

## Results

The collected data on movement behaviors in the studied group revealed that during the monitored 7-day period, sedentary behaviors (SB) accounted for an average of 8 h and 34 min per day, while the mean number of steps taken was slightly over 8,000 steps per day. The results of the Shapiro–Wilk test, which assessed the assumption of normality for these two primary variables related to movement behaviors, indicated that the distribution of time spent sitting or lying down met the normality assumption. However, the distribution of the number of steps was slightly skewed (Table 2).

**Table 2:** Descriptive statistics of the studied variables with the Shapiro–Wilk test (*N* = 173).

BehavioralMetrics	*M*	Mdn	SD	Skewness	Kurt.	Min.	Max.	W	*p*
SB	8:34:26	8:37:27	1:37:32	0.19	0.21	4:43:09	13:36:24	0.99	0.352
Steps	8007.42	7511.14	3526.66	1.04	1.89	642.86	21502.00	0.94	<0.001*

* Statistical significance.

This result allowed for the analysis of correlations between self-rated health, socio-demographic factors, and movement behaviors using both parametric and non-parametric tests.

**Figure 1:**
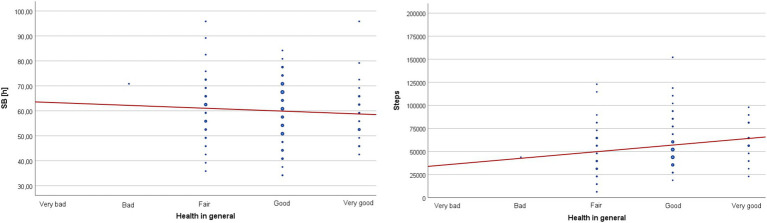
Scatter plot with a fitted line for the correlation between self-rated health and cumulative sedentary behavior time (SB) as well as cumulative step count (Steps) from the entire monitoring period.

The results of the analysis confirmed a significant positive correlation between self-rated health and the number of steps taken during the monitoring period. This indicates that as self-rated health increased, participants took a greater number of steps. However, the observed correlation was weak (rho < 0.30) – Table 3 and Figure 1.

**Table 3:** Correlation between self-rated health and movement behaviors of the studied individuals (*N* = 173).

BehavioralMetrics	Health in general	
	Spearman’s rho	*p*
SB	−0.07	0.333
Steps	0.23	0.002*

* Statistical significance.

Subsequently, the occurrence and strength of correlations between selected socio-demographic factors characterizing the participants – such as gender, age, area and type of housing, household size, marital status, and occupational activity – and variables characterizing a sedentary lifestyle were examined.

The analysis revealed a significant difference between women and men in terms of sedentary behaviors (SB). Compared to men, women spent significantly less time sitting or lying down and also performed noticeably more steps; however, the correlation for this variable was not statistically significant – Table 4.

**Table 4:** Correlations between movement behaviors and participants’ gender (*N* = 173).

BehavioralMetrics	Men (*n* = 102)			Women (*n* = 71)					
	Mean rank	*M*	SD	Mean rank	*M*	SD	*Z*	*p*	*η*2
SB	80.69	8:20:58	01:29:44	96.06	8:53:48	01:45:25	−1.99	0.047*	0.02
Steps	81.01	7690.74	3436.72	95.61	8462.35	3628.02	−1.89	0.059	0.02

* Statistical significance.

The analysis of the correlation between age categories and the prevalence of sedentary behaviors showed that significant differences occurred only in the number of steps taken – Table 5.

**Table 5:** Correlations between movement behaviors and age categories (*N* = 173).

BehavioralMetrics	Age groups	Mean rank	*M*	SD	H(2)	*p*	*η* ^2^
SB	18–34 years (*n* = 53)	95.97	8:49:19	1:30:49	6,02	0.111	0.02
	35–49 years (*n* = 41)	72.88	8:06:17	1:32:01			
	50–64 years (*n* = 44)	83.27	8:28:54	1:50:20			
	65 years and older (*n* = 35)	94.64	8:51:52	1:32:12			
Steps	18–34 years (*n* = 53)	93.47	8413.56	3228.90	21.33	<0.001*	0.11
	35–49 years (*n* = 41)	109.67	9494.57	3807.49			
	50–64 years (*n* = 44)	80.78	7738.93	3590.92			
	65 years and older (*n* = 35)	58.43	5887.82	2528.50			

* Statistical significance.

To assess the significance of the detected differences, the post-hoc Dunn test was conducted. The results showed that significant differences within age groups occurred between: (a) individuals from the youngest age group (18–34 years) and the oldest group (> 64 years), (b) individuals aged 35–49 years and the oldest group, (c) individuals aged 35–49 years and those aged 50–64 years, and (d) individuals aged 50–64 years and the oldest age group. Participants aged 35–49 took the most steps, while those aged over 64 took the fewest steps during the monitoring period – Table 6.

**Table 6:** Results of the *post-hoc* Dunn test comparing age categories pairwise.

BehavioralMetrics	18–34 years vs. 65 years and older	18–34 years vs. 50–64 years	18–34 years vs. 35–49 years	35–49 lat vs. 65 years and older	35–49 years vs. 50–64 years	50–64 years vs. 65 years and older
Steps	−35.02***	−12.69	−16.20	−51.21***	−28.89**	−22.33*

Statistical significance: * *p* < 0.050; ** *p* < 0.010; *** *p* < 0.001.

The analysis of the correlation between area of residence and variables characterizing a sedentary lifestyle considered two most represented socio-demographic categories: (a) living in urban areas, and (b) living in rural areas. The analysis was conducted using the non-parametric Mann–Whitney test – Table 7.

**Table 7:** Correlations between individuals living in rural areas and those living in urban areas in terms of movement behaviors (*N* = 172).

BehavioralMetrics	Urban areas (*n* = 135)			Rural areas (*n* = 37)			*Z*	*p*	*η* ^2^
	Mean rank	*M*	SD	Mean rank	*M*	SD			
SB	85.63	8:33:11	1:36:56	89.69	8:41:09	1:41:09	−0.44	0.660	<0.01
Steps	88.13	8112.81	3689.36	80.54	7529.02	2854.21	−0.82	0.411	<0.01

**Figure 2:**
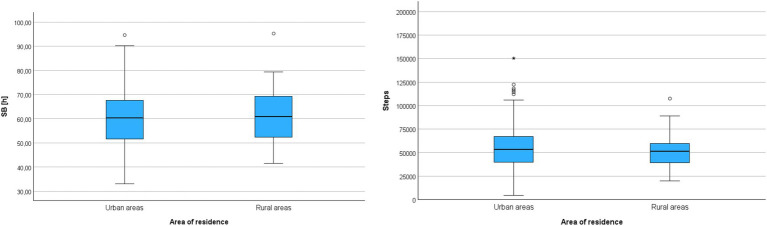
Cumulative sedentary behavior time (SB) and cumulative step count (Steps) during the entire monitoring period in groups differentiated by area of residence.

The analysis did not reveal statistically significant differences between the compared groups in terms of sitting or lying time (SB) or the number of steps. Thus, regardless of whether participants lived in rural or urban areas, they exhibited a similar profile of movement behaviors – Figure 2.

In the next analysis, it was examined whether the type of housing differentiated participants in terms of movement behaviors. The correlation analysis between residents of single-family houses and residents of apartment buildings was conducted using the Mann–Whitney test – Table 8.

**Table 8:** Correlations between groups differentiated by type of housing and movement behaviors (*N* = 173).

BehavioralMetrics	Apartment (*n* = 73)			House (*n* = 100)			*Z*	*p*	*η* ^2^
	Mean rank	*M*	SD	Mean rank	*M*	SD			
SB	84.68	8:30:17	1:36:07	88.70	8:37:28	1:38:55	−0.52	0.602	<0.01
Steps	93.45	8665.44	4000.84	82.30	7527.06	3068.33	−1.45	0.148	0.01

**Figure 3:**
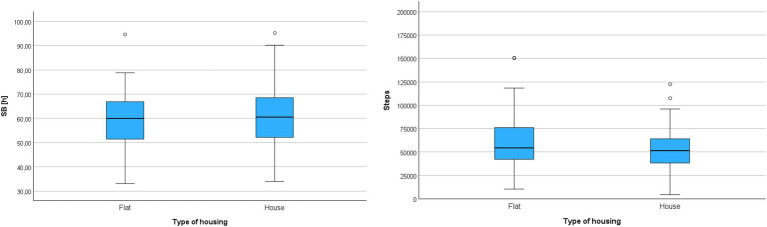
Cumulative sedentary behavior time (SB) and cumulative step count (Steps) during the entire monitoring period in groups differentiated by type of housing.

The analysis did not reveal statistically significant differences between the compared groups. Regardless of whether participants lived in apartment buildings or single-family houses, they exhibited a similar weekly sedentary behavior time (SB) and a similar weekly step count (Steps) – Figure 3.

**Figure 4:**
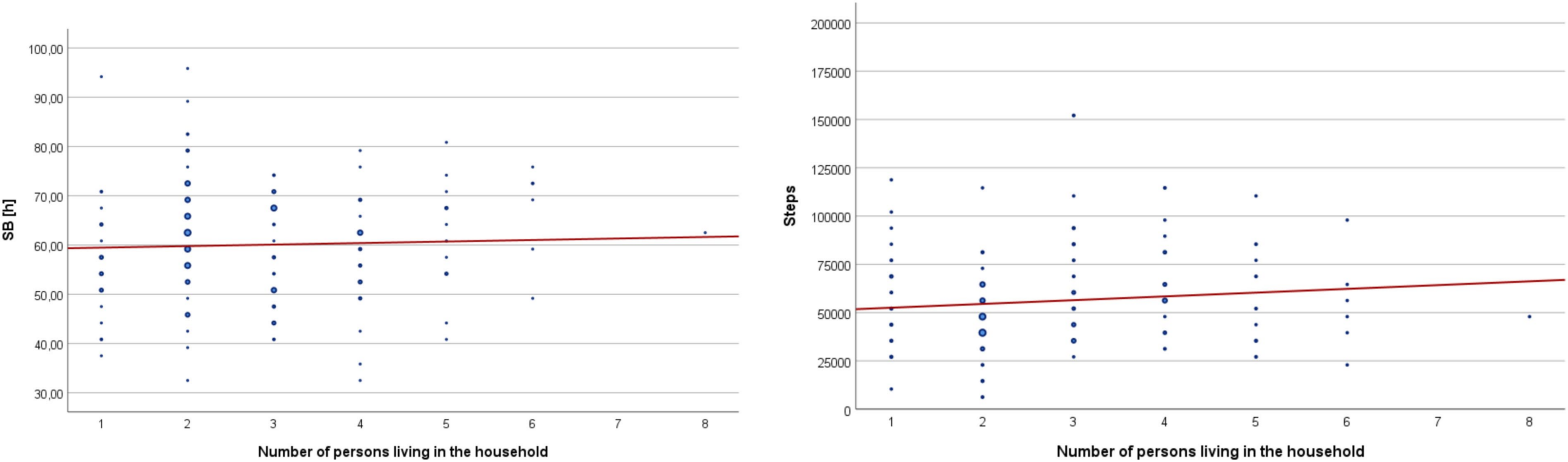
Cumulative sedentary behavior time (SB) and cumulative step count (Steps) during the entire monitoring period in groups differentiated by the number of household members living together.

The number of people living with respondents in the same household is a socio-demographic variable that significantly differentiated the studied group. Spearman’s rho correlation analysis for homogeneous subgroups did not reveal statistically significant correlations between the defined groups and movement behaviors – Table 9 and Figure 4.

**Table 9:** Correlations between the number of household members and movement behaviors of participants (*N* = 173).

BehavioralMetrics	Number of persons living in the household	
	Spearman’s rho	*p*
SB	0.04	0.625
Steps	0.13	0.084

**Figure 5:**
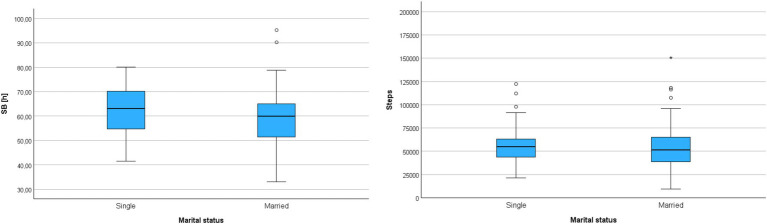
Cumulative sedentary behavior time (SB) and cumulative step count (Steps) during the entire monitoring period in groups differentiated by marital status.

Another socio-demographic variable included in the correlational study was the marital status of the participants. The statistical analysis considering this characteristic revealed a significant difference between the compared groups, but only in terms of sitting or lying time (SB). It was found that individuals who were not in a relationship (single, divorced, widowed) had significantly longer weekly sitting or lying time compared to those who were married. However, it should be noted that the observed effect was weak (0.06 > *η*^2^) – Table 10 and Figure 5.

**Table 10:** Correlations between marital status and movement behaviors of participants (*N* = 141).

BehavioralMetrics	Single (*n* = 50)			Married (*n* = 91)			*Z*	*p*	*η* ^2^
	Mean rank	*M*	SD	Mean rank	*M*	SD			
SB	81.57	8:56:48	1:28:31	65.19	8:22:21	1:34:52	−2.28	0.023*	0.04
Steps	75.04	8156.81	3004.90	68.78	7831.56	3481.93	−0.87	0.384	<0.01

* Statistical significance.

The last socio-demographic variable included in the analyses differentiating the participants was the current employment status, which was characterized by three differentiating variables: (a) white-collar workers, (b) unemployed, and (c) students. The correlation analysis was conducted using the Kruskal-Wallis test – Table 11.

**Table 11:** Correlations between groups with different employment statuses and movement behaviors (*N* = 169).

BehavioralMetrics		Mean rank	*M*	SD	H(2)	*p*	*η* ^2^
SB	White-collar worker (*n* = 87)	78.02	8:23:27	1:37:21	4.89	0.087	0.02
	Unemployed (*n* = 43)	86.67	8:39:01	1:38:46			
	Student (*n* = 39)	98.73	8:56:45	1:32:16			
Steps	White-collar worker (*n* = 87)	98.30	62842.79	26753.95	18.97	<0.001*	0.10
	Unemployed (*n* = 43)	58.58	42802.86	16944.14			
	Student (*n* = 39)	84.46	56057.03	21884.81			

* Statistical significance.

**Figure 6:**
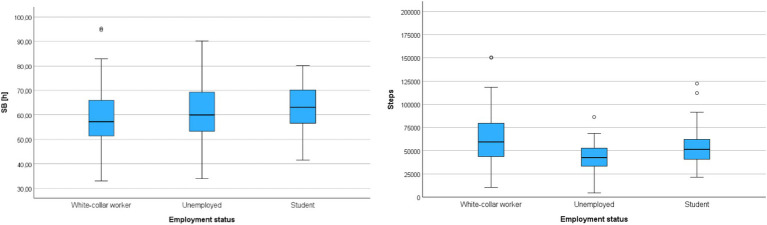
Cumulative sedentary behavior time (SB) and cumulative step count (Steps) during the entire monitoring period in groups differentiated by employment status.

The analysis revealed a significant difference between the compared groups only in terms of the number of steps, with a moderate strength of correlation (0.06 < *η*^2^ < 0.14). Additionally, the post-hoc Dunn test showed that white-collar workers had a significantly higher weekly step count compared to unemployed individuals (*Z* = 4.35; *p* < 0.001) – Figure 6.

The final stage of the statistical analysis involved verifying models explaining movement behaviors (SB, Steps) based on data on self-rated health and socio-demographic factors characterizing the participants. A linear regression analysis was performed, with sitting or lying time (SB) and step count (Steps) as the dependent variables, and self-rated health, gender, age, employment status, marital status, household size, and area of residence and type of housing as the independent variables – Table 12.

**Table 12:** Regression models explaining movement behaviors (*N* = 146).

BehavioralMetrics		*B*	SE	Beta	*t*	*p*
SB	*F*(8;132) = 1.35; *p* = 0.226; *R*^2^*adj*. = 0.019					
	(Constant)	237570.95	29981.16		7.92	<0.001*
	Gender	10896.96	6823.56	0.14	1.60	0.113
	Age	269.56	326.47	0.13	0.83	0.410
	Marital status	−17695.08	12319.50	−0.22	−1.44	0.153
	Employment status	−8368.68	7856.38	−0.11	−1.07	0.289
	Household size	1848.50	2968.93	0.06	0.62	0.535
	Area of residence	790.10	8983.08	0.01	0.09	0.930
	Type of housing	2597.81	7499.19	0.03	0.35	0.730
	Health in general	−4207.81	5290.93	−0.07	−0.80	0.428
Steps	*F*(8;132) = 3.34; *p* = 0.002; *R*^2^*adj*. = 0.118					
	(Constant)	60751.80	16735.34		3.63	<0.001*
	Gender	7693.05	3808.88	0.17	2.02	0.045*
	Age	−330.83	182.24	−0.26	−1.82	0.072
	Marital status	2929.95	6876.68	0.06	0.43	0.671
	Employment status	9192.77	4385.39	0.20	2.10	0.038*
	Household size	1119.25	1657.24	0.06	0.68	0.501
	Area of residence	−1799.92	5014.31	−0.03	−0.36	0.720
	Type of housing	−6780.74	4186.01	−0.14	−1.62	0.108
	Health in general	2083.57	2953.37	0.06	0.71	0.482

* Statistical significance.

The analysis revealed that the model explaining sitting and/or lying time is not well-fitted to the data, accounting for only 1.9% of the variance in SB. This indicates that it is not possible to reliably predict such behaviors based on gender, age, self-rated health, employment status, marital status, household size as well as area of residence and type of housing. However, the results showed that in the case of step count, which serves as an indirect measure of sedentary (or active) tendencies, employment status is a statistically significant predictor. For this variable, the model explains 11.8% of the variance. Beta coefficient values associated with employment status and gender indicated that these factors predispose individuals to perform a higher number of steps per week.

## Discussion

The results obtained in our study showed that the prevalence of sedentary behaviors among adult Poles residing in eastern Poland was very high. For more than 50% of the respondents, the time allocated to sedentary behaviors during the monitored period exceeded the cut-off proposed by Chau et al. (32), i.e., 8 h per day. Beyond this threshold – if physical activity is not accounted for – each additional hour spent sitting is associated with an 8% increase in the risk of all-cause mortality. The results of our study are more than twice as high as the data reported for the Polish population in both the Eurobarometer 412 (33) and the Eurobarometer (19) studies. However, these data only serve as a point of reference for our findings, which, although not obtained through a representative population-based study, may reflect a pattern of sedentary behaviors close to reality. This is due to the objective measurement methods used and the good representation of gender and age, at least for a large segment of the population in eastern Poland.

Similar results were obtained by Nicolson et al. (34) in a study of the Irish population, where sedentary behaviors also predominated, with overall sitting time exceeding 7.5 h per day. On the other hand, Ferrari et al. (35) reported even higher levels of sedentary behaviors among adolescents and adults (aged 15–65 years) in Latin American countries, averaging 9.53 h per day (ranging from 9.23 h in Chile to 9.95 h in Peru).

The lack of significant associations between sedentary behaviors and the place or type of residence in our study may be attributed, on the one hand, to sampling errors, or - more likely - to the homogeneity of the built environment in the study area, as demonstrated in numerous original studies (36–38) and a review paper (39). Our findings enable the formulation of preliminary hypotheses, such as that the place of residence (urban vs. rural) and the type of residence are not correlated with sedentary behaviors in communities inhabiting low-urbanized areas (e.g., eastern Poland), with sociodemographic correlates being the primary differentiating factors.

The correlation between sedentary behaviors and socio-demographic factors was not clearly confirmed in our study. Significant correlations were detected only for marital status and current employment status (employed vs. unemployed). Regarding marital status, being single was associated with prolonged periods of sedentary behaviors, while in the case of employment status, unemployed individuals performed significantly fewer steps during the monitoring period. However, it is important to emphasize that the strength of these correlations was weak and moderate, respectively. The results confirm the regularities identified in the HAPA model that self-efficacy in performance and maintenance and performance expectations had little to moderate impact on health behaviors, including those related to healthy movement behaviors (40, 41).

One of the correlates of a sedentary lifestyle is gender and age; however, researchers’ positions on this matter are not unanimous. Studies conducted in Germany (42) and the Netherlands (43) indicate that men are the group in which sedentary behaviors dominate. Conversely, a detailed multiple correspondence analysis by Meneguci et al. (44), conducted on a population of older adult Brazilians, revealed the opposite correlation – women and individuals aged over 70 years were the groups characterized by higher sitting time. Other studies conducted on populations from Latin American countries, however, showed that sitting time increases with age, with the lowest levels observed in the middle-age group (25–64 years). Notably, the significant gender difference in these populations favored women – women spend less time sitting.

Our study confirmed the correlations observed in the German and Dutch populations regarding the relationship between age and sedentary behaviors. However, we did not find significant differences related to gender. The results of our research also demonstrated that the determinants of sedentary behaviors in adults are highly complex, with the impact of socio-demographic factors being ambiguous and limited. This aligns with the findings of the SOS framework developed by the DEDIPAK KH team, which identified as many as 234 factors associated with sedentary behaviors in adults and the older adult (45). Nevertheless, the results obtained in groups differentiated by marital and occupational status are consistent with the study conducted on the adult Japanese population (46).

One of the significant correlates of sedentary behaviors in adults is self-rated health. Our findings partially confirmed these correlations, indicating that individuals with a higher self-assessment of their health were more active, taking more steps during the monitoring period (*p* < 0.001). The scientific literature on self-rated health in the context of sedentary behaviors is limited, and the available findings are inconsistent. In the study by Peltzer et al. (47), based on research involving young adults (18–25 years), no significant associations between overall health status and sedentary behaviors (SB) were observed, with positive correlations only reported for individuals with depression. Conversely, in the study of Canadian older adults (60–79 years), significant correlations were identified for both low (*p* < 0.01) and high (*p* < 0.05) self-rated health (48). According to Meneguci et al. (44), interventions aimed at reducing sedentary time could be effective in achieving and maintaining good health by improving self-rated health.

The regression models developed based on the data obtained in our study, due to the very low (1.9%) and low (11.8%) levels of variance explained for SB and Steps, respectively, did not allow for a clear prediction of movement behaviors within homogeneous subgroups defined by socio-demographic factors and subjective overall health assessment. Consequently, we cannot identify any factor considered as causative, i.e., determining sedentary behaviors. This result indicates the need for more population-based studies aimed at finding predictors of SB. The variance for the number of steps taken is a significant indication of the factors included in our study. The only statistically significant predictor associated with reducing sedentary behaviors in the studied cohort was occupational activity – specifically office work. The result like this may suggest that individuals engaged in intellectual work – despite spending the majority of their working hours sitting – are at a lower risk of sedentary behaviors compared to non-working individuals and students (49).

The socio-demographic correlates identified in our study should be considered when designing interventional measures, including educational campaigns and the monitoring of movement behaviors using accelerometry. These elements should be integrated into local health programs, along with an evaluation of the effectiveness of the implemented interventions.

### Advantages and limitations

A strength of our study was the objective assessment of health behaviors, whereas most available studies conducted on the Polish population are based on surveys and rely on self-reported sedentary behaviors (SB). However, this study also has some limitations. Firstly, it was an observational study, which precludes drawing causal conclusions. Additionally, due to the voluntary nature of participation, the study may have primarily attracted more motivated and active individuals as well as participants with a desire to obtain an accurate assessment of their physical activity levels and health-related parameters. These factors may have influenced the results and limited the generalizability of the findings. Consequently, the results should be considered preliminary and require further validation in larger, representative samples.

## Conclusion

The study results indicate that employment status and gender are the strongest socio-demographic correlates of sedentary behaviors among adult residents of eastern Poland. Unemployment (including unemployed individuals, students, retirees, and those dependent on others) emerged as the most significant correlate of prolonged time spent sitting and a lower daily step count. Regarding gender, men exhibited more unfavorable patterns of physical activity compared to women.

Additionally, it was observed that middle-aged individuals in marital relationships were the least prone to sedentary behaviors. The influence of other socio-demographic factors, such as area of residence, type of housing, or household size, was limited. Even in cases where statistically significant correlations were noted, their strength was minimal.

The results emphasize the need for detailed research to better understand the determinants of sedentary behaviors. At the same time, they highlight the importance of designing intervention strategies aimed at reducing sedentary lifestyles and promoting physical activity, tailored to the specific needs of different demographic groups.

## Data Availability

The datasets presented in this article are not readily available because the database on which the article is based is held by the corresponding author and can be made available upon reasonable request, subject to legal restrictions. Requests to access the datasets should be directed to m.stelmach@dyd.akademiabialska.pl.
